# Giant Electric Field Enhancement in Split Ring Resonators Featuring Nanometer-Sized Gaps

**DOI:** 10.1038/srep08051

**Published:** 2015-01-27

**Authors:** S. Bagiante, F. Enderli, J. Fabiańska, H. Sigg, T. Feurer

**Affiliations:** 1 Laboratory of Micro- and Nanotechnology Paul Scherrer Institute, Villigen 5232, Switzerland; 2 Institute of Applied Physics University of Bern, Bern 3012, Sidlerstrasse 5, Switzerland

## Abstract

Today's pulsed THz sources enable us to excite, probe, and coherently control the vibrational or rotational dynamics of organic and inorganic materials on ultrafast time scales. Driven by standard laser sources THz electric field strengths of up to several MVm^−1^ have been reported and in order to reach even higher electric field strengths the use of dedicated electric field enhancement structures has been proposed. Here, we demonstrate resonant electric field enhancement structures, which concentrate the incident electric field in sub-diffraction size volumes and show an electric field enhancement as high as ~14,000 at 50 GHz. These values have been confirmed through a combination of near-field imaging experiments and electromagnetic simulations.

Over the past 20 years, continuous progress in Terahertz (THz) technologies has facilitated numerous breakthroughs in scientific and industrial research^1^,^2^,^3^. Mostly through linear time-domain THz spectroscopy we have witnessed a marked increase in THz research on molecules^4^, biomolecules^5^, liquids^6^, semiconductors^5^, superconductors^7^, crystals or complex materials^8^. Moreover, THz spectroscopy forms the basis for stand-off detection of hidden chemicals^9^,^10^; in comparison to visible or infrared radiation, THz frequencies can penetrate into organic materials such as skin, plastics, cloths, or paper products and have thus become indispensable in security applications. An additional benefit is that THz radiation, in contrast to X-rays, does not cause any damage associated with ionization^11^.

Despite all progress, the generation of THz pulses with high peak electric fields is still rather limited, mostly due to technological constraints such as available laser power and low conversion efficiencies^5^,^12^. The highest THz powers are currently available from large-scale electron particle accelerators^13^, but also tabletop sources show similar powers when using, for example, large area photoconductive switches, frequency mixing in laser generated plasmas, or optical rectification in nonlinear crystals^14^,^15^,^16^,^17^,^18^. In order to extend THz experiments into the nonlinear regime the quantity to be optimized is the electric field strength *E*^19^. It is linked to the THz pulse energy *Q* through the relation 
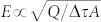
 where Δ*τ* is the temporal pulse duration and *A* the beam area. The available pulse energy is naturally limited by the THz system at hand and the pulses are often already single-cycle, so that the electric field strength cannot be increased by adjusting the parameters *Q* and Δ*τ*. The beam area can be minimized through tight focusing, however, the diffraction limit imposes a lower boundary on *A*.

Diffraction limited focusing can be overcome, for example, by using suitable metallic structures which act as antennas efficiently collecting the incident radiation and concentrating it in a small sub-wavelength sized volume. In the process, part of the incident radiation is typically converted to charge density oscillations, which in turn generate an enhanced and localized electric field distribution on a length scale well beyond the diffraction limit^20^,^21^,^22^,^23^. At visible wavelengths this concept has turned into a key element for example for single-molecule spectroscopy, nano-imaging, or extreme nonlinear optics^24^,^25^,^26^. Recently, Seo et al.^21^ and Blanchard et al.^22^ have demonstrated that similar concepts are applicable in the THz regime. By illuminating a dipole antenna in form of a nano-slit Seo and coworkers demonstrated strong electric field localization. For a 70 nm wide nano-slit and a frequency of 0.1 THz the electric field enhancement was on the order of 1,000^21^,^27^.

Here, we report on a novel antenna design, i.e. a split ring resonator featuring a nanometer sized gap, which extends into the inner part of the split ring resonator. Such structures, as outlined below, show promise for extremely high THz electric field enhancement at their resonance frequencies. These frequencies can be easily tuned throughout the entire THz spectrum by changing the structure's dimensions. Since there exists no detection modality with sufficient spatial resolution to map out the in-gap electric field we have to resort to indirect methods. Based on near-field measurements of the oscillating charge distribution on the split ring resonator we calibrate detailed finite element simulations to derive the THz electric field enhancement and THz electric field amplitude in the gap. Throughout the manuscript the electric field enhancement as well as the in-gap electric field are averaged over the gap volume and may be lower or higher at specific points within the gap. At the lowest order resonance of the specific design we find that the THz electric field enhancement reaches 14,300 at 50 GHz for a 100 nm wide gap and is the highest reported to date in this frequency regime. The corresponding maximum *|E|*^*2*^ and *|E|*^*4*^ enhancement factors are 2·10^8^ and 4·10^16^, respectively, exceeding values typically observed in surface enhanced Raman spectroscopy^28^. Therefore, such antenna structures are an excellent platform for nonlinear THz science, small THz signal detection, or few molecule and nano-particle detection. By irradiating the structures with THz pulses from a standard femtosecond oscillator driven spectroscopy source we find in-gap electric field strengths of approximately 80 kVm^−1^; such electric field strength are usually only achieved with amplified femtosecond laser systems.

## Results

**Figure 1:**
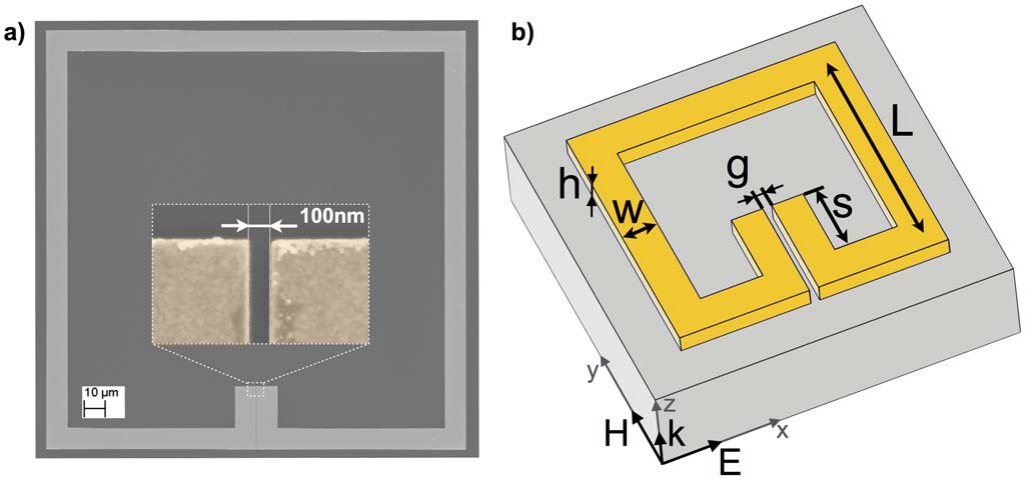
(a) SEM image of the split ring resonator and a close-up view, which shows part of the 100 nm wide gap region. (b) Schematic illustration of the split ring resonator with all relevant dimensions, the *(E, H, k)* triad of the incident THz field, *k* refers to the wave vector, and the coordinate system *(x,y,z)*.

Figure 1a) shows an image of a split ring resonator with extended capacitive faces and a close-up view of the gap recorded with a scanning electron microscope. The length of the extended capacitive faces can be adjusted to control the gap volume and therefore the interaction volume without changing the area covered by the split ring resonator^29^,^30^. The substrate is high resistivity silicon and the relevant dimensions of the gold structures investigated hereafter are *L* = 200 μm, *w* *= * 10 μm, *s* *= * 20 μm, and *h* *= * 60 nm (see Figure 1b). Three different isolated split ring resonators were fabricated featuring gap widths of *g* *= * 100 nm, 500 nm, and 970 nm, respectively. The total length of the unfolded ring was chosen such as to find the fundamental and the third resonance at approximately ν_1_ = 60 GHz and ν_3_ = 180 GHz. Note that even order resonances are not excited here. For these parameters the structure size is well matched to the 20 μm spatial resolution of the THz near-field imaging setup^31^,^32^,^33^.

At the fundamental resonance, the incident THz radiation induces a current flow on the metal surface that leads to an accumulation of charge carriers around the gap region. This capacitive charging in turn results in an in-gap electric field enhancement by orders of magnitude. The electromagnetic energy stored in the SRR and, thus, the amount of charge carriers accumulating in the gap region is finite. For increasing gap length, the charge accumulations are therefore distributed over a larger distance, so that the field enhancement is found to decrease^29^. For comparison, the magnetic field enhancement is typically one to two orders of magnitude smaller than the electric field enhancement. At higher order resonances, the induced current flow shows a more complex pattern and typically results in somewhat lower electric field enhancement values^34^. Since no THz detection system has the spatial resolution required to directly measure, let alone map out, the electric field distribution in the gap volume, we resort to quantify the charge distribution in the split ring resonator, which is responsible for the electric field enhancement in the gap. For this we use a THz near-field setup (for more experimental details see also supplementary information) configured to measure the electric field component normal to the plane containing the split ring resonator, which is directly proportional to the charge distribution.

**Figure 2:**
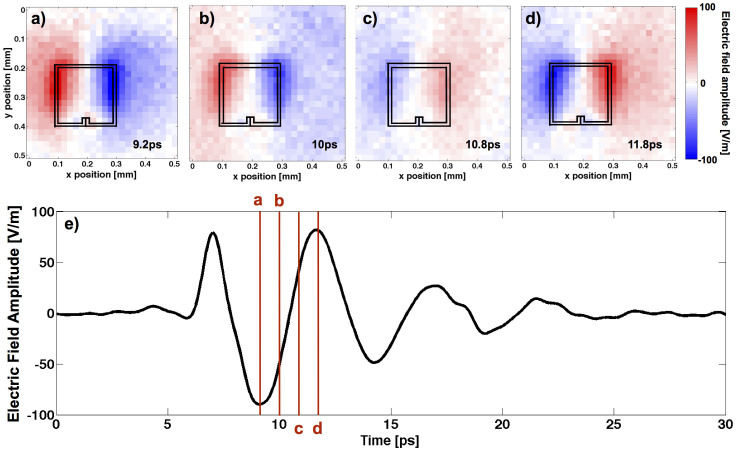
Measured time dependent out-of-plane electric field for a split ring resonator with a 500 nm wide gap. The intensity graphs show the electric field distribution *E_z_(x,y,t_j_)* at successive times *t_j_* = 9.2 ps (a), 10 ps (b), 10.8 ps (c), and 11.8 ps (d), respectively. The time dependent electric field measured at the top right corner of the structure is shown in e) and the *t_j_* are marked by red vertical lines. The supplementary online material contains a movie showing the full time dependent electric field distribution *E_z_(x,y,t)* from which Figs. (a)–(d) were extracted.

Exemplarily, we discuss the results for the split ring resonator with a 500 nm wide gap. The incident single-cycle THz pulses cover a spectral range from 10 GHz to about 1.5 THz with the peak at approximately 0.32 THz. They propagate along the *z*-axis and the sample is positioned at the image plane of the THz source. There, the THz electric field is polarized primarily along the *x*-axis and is parallel to the side of the split ring resonator which contains the gap. The *(E, H, k)* triad – where *E* is the electric and *H* the magnetic field and *k* the wave vector – of the incident THz pulse is indicated in Fig. 1b). Figure 2e) shows the measured time dependent out-of-plane electric field *E_z_(t)* averaged over 9 pixels around the top right corner of the split ring resonator. Note that the incident THz pulse has a negligible longitudinal electric field component and the measured electric field is solely due to the THz-induced charge oscillations. Figures 2a) to d) show snapshots of the measured out-of-plane electric field distribution *E_z_(x,y,t_j_)* at successive times *t_j_* = 9.2 ps, 10 ps, 10.8 ps, and 11.8 ps as marked by the vertical red lines in Fig. 2e). The color scale indicates the absolutely calibrated electric field (see supplementary information).

**Figure 3:**
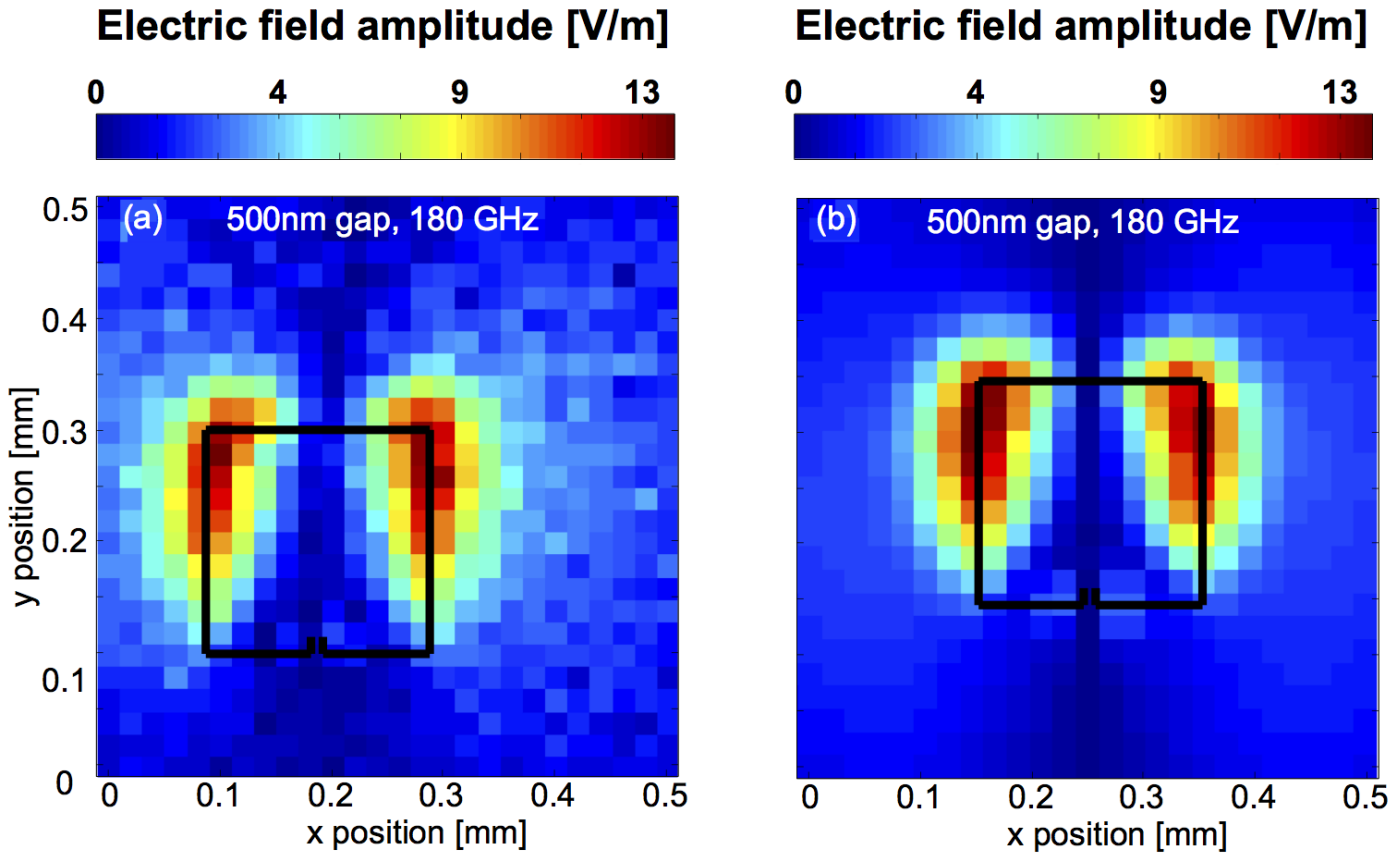
Intensity graphs of the measured a) and the simulated b) out-of-plane electric field distribution |*E_z_(x,y,ν_3_)*| at the third resonance.

From the calibrated temporal electric field distribution *E_z_(x,y,t)* we calculate the spectral electric field distribution *E_z_(x,y,ν)* by Fourier transforming the time dependent electric field at every position *(x,y)*. The intensity graph in Fig. 3a) shows the measured electric field distribution |*E_z_(x,y,ν_3_)|* at the third resonance, i.e. at *ν_3_* = 180 GHz. The signal at the first resonance is close to the noise floor and not considered here.

Comparing the measured (Fig. 3a) to the simulated (Fig. 3b) spatial distribution of the out-of-plane electric field |*E_z_(x,y,ν_3_)*| shows excellent agreement. The simulations are performed in frequency-domain and in three spatial dimensions using a commercial software package (COMSOL Multiphysics)^35^. The simulations take into account the substrate on which the split ring resonator structure is fabricated as well as the electro-optic detection crystal, which is an integral part of the near-field detection system. The only free parameter in the simulations is the electric field strength of the incident THz radiation, which we determine from a separate measurement of *E_x_(t)* with no split ring resonator on the substrate. The simulations not only yield the out-of-plane electric field distribution but any other relevant quantity, most importantly the electric field distribution and the electric field enhancement in the gap where the measurements, due to the limited spatial resolution, cannot reveal any information. That is, we experimentally verify that the simulations accurately reproduce the spatially resolved out-of-plane electric field at the third resonance (*ν_3_* = 180 GHz), and thus the charge distribution on the split ring resonator which is responsible for the capacitive charging of the gap, and then extract the in-gap electric field and field enhancement from the simulations.

**Figure 4:**
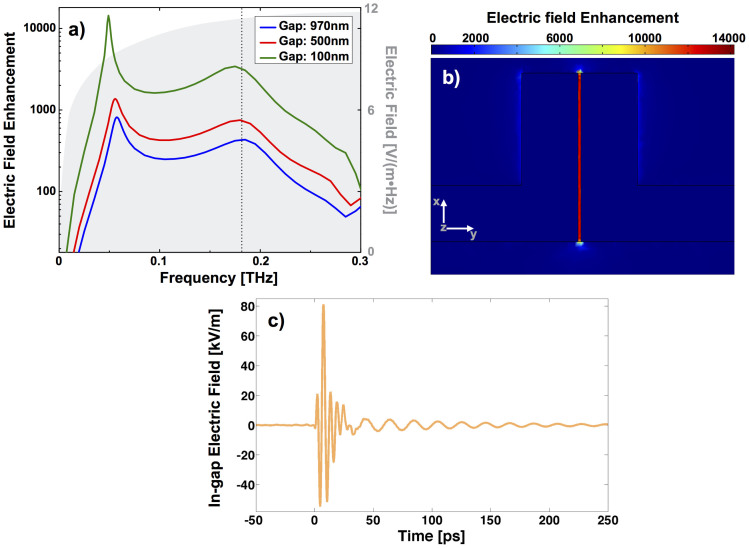
(a) Absolute value of the electric field enhancement in the gap as a function of frequency for the three gap widths 100 nm, 500 nm, and 970 nm, respectively. Absolute electric field amplitude of the incident THz spectrum in gray. (b) Electric field enhancement distribution in a 500 nm gap SRR at 56 GHz. (c) In-gap electric field strength versus time for the 100 nm wide gap.

Figure 4a) shows the simulated in-gap electric field enhancement as a function of frequency for all three gap widths examined. The black vertical dashed line marks the calibration points for all three gap widths at the third order resonance frequencies *ν*_3_. For a 100 nm wide gap the maximum electric field enhancement is as high as 14300 at the fundamental resonance and 3400 at the third order resonance. As mentioned above the electric field enhancement at the individual resonances differ because of the different character of the charge distribution on the split ring structure. At the lowest order resonance the charge is known to accumulate predominantly around the gap. Conversely, at the third order resonance, the charges accumulate at the two corners opposite to the gap^36^, therefore the electric field enhancement is lower. The spatial in-gap distribution of the electric field enhancement in the xy plane (at z = h/2 = 30 nm, g = 500 nm, *v_1_* = 56 GHz) is shown in Figure 4b). Except some variations at the edges, it is relatively homogeneous in the entire region.

Figure 4c) shows the calibrated in-gap electric field as obtained by multiplying the incident THz spectrum (gray shaded area in Fig. 4a)) with the complex-valued electric field enhancement for the 100 nm wide gap. The phase associated to the absolute value of the electric field enhancement shown in Fig. 4a) was calculated by means of Kramer-Kronig's relation. The in-gap THz electric field has two distinct frequency components, i.e. a short lived at *ν_3_* = 175 GHz (broad line width) and a long lived at *ν_3_* = 50 GHz (narrow line width), and reaches peak values on the order of 80 kVm^−1^. The results suggest that even a conventional tabletop THz source, as it is used in many THz time domain spectrometers, combined with such a split ring resonator can result in electric field amplitudes as high as several hundreds of kVm^−1^. A more powerful THz source will yield electric fields sufficiently high for many nonlinear THz effects, for example in semiconductors.

We would like to point out that the dimensions of the split ring resonators used here have been chosen to allow for sophisticated near-field THz imaging and not for achieving the highest possible electric field strengths. For such purpose one would aim for a better overlap between the incident THz spectrum and the electric field enhancement curve. Although available fabrication techniques permit narrower gap widths (the smallest gap width is most likely limited by the Thomas-Fermi screening length when non-local effects have to be considered; this occurs below 1 nm), and thus even higher electric field enhancements, these are not necessarily advantageous as they result in a smaller and more difficult to access interaction volume. Therefore, a compromise should be found between electric field enhancement and interaction volume.

## Discussion

Through a combination of THz near-field imaging measurements and sophisticated electromagnetic simulations we have demonstrated giant electric field enhancement in split ring resonators featuring a nano-gap. Electric field enhancement values as high as ~14,000 and ~3,400 were observed for a 100 nm wide gap with the resonance frequencies at 50 GHz and 175 GHz. At 50 G*Hz t*he corresponding maximum *|E|*^2^ and *|E|*^4^ enhancement factors are 2·10^8^ and 4·10^16^. Through a judicious choice of the split ring resonator's dimensions the resonances can be easily tuned throughout the entire THz region, and thus matched to vibrational or rotational resonances in molecules or phonon resonances in solids. Moreover, their width can be adjusted by exploiting strong couplings to other resonant structures as shown for example in reference^37^. These results represent an important step towards sub-diffraction limited THz focusing, THz electric field enhancement, and their use in linear and nonlinear THz experiments. Using appropriate resonators, nonlinear experiments starting from even common laboratory THz sources are conceivable.

## Author Contributions

F.E. and S.B. performed the experiments; S.B. fabricated the sample; J.F. and F.E. performed the simulations; S.B., H.S. and T.F. wrote the manuscript; all authors discussed the results and contributed to the manuscript.

## Supplementary Material

Supplementary Information Supplementary information

Supplementary Information Time dependent electric field distribution
