# Supplementary material for: Giant Electric Field Enhancement in Split Ring Resonators Featuring Nanometer-Sized Gaps
Source: Sci Rep. 2015 Jan 27;5:8051. doi: 10.1038/srep08051 (PMC4306966; doi:10.1038/srep08051)
Supplement: Supplementary Information [file srep08051-s1.pdf]

Giant Electric Field Enhancement in Split Ring Resonators Featuring Nanometer-Sized Gaps

S. Bagiante[1, 2], F. Enderli[1, 2], J. Fabiańska[2], H. Sigg[1] and T. Feurer[2]

[1] Laboratory of Micro- and Nanotechnology Paul Scherrer Institute, Villigen 5232, Switzerland
[2] Institute of Applied Physics University of Bern, Bern 3012, Sidlerstrasse 5, Switzerland

Supplementary information

Sample fabrication: Single split ring resonators with different gap widths were fabricated on 2 cm by 2 cm large and 550 μm thick high resistivity silicon wafers (ρ = 18000 to 50000 Ωcm). Using electron-beam lithography and electron-beam evaporation for metal deposition, the planar structures were defined. After depositing 3 nm of chromium and 60 nm of gold all excess metal was removed with the lift-off technique.

Experimental setup: The THz near-field microscope is powered by a femtosecond fiber laser (Femto Fiber Pro, Toptica). It has a repetition rate of 82 MHz, a pulse duration of 80 fs, and an average power of 138 mW at a wavelength of 800 nm. The laser pulses are split in two time-delayed replicas with about 50 mW of the available laser power being used for THz generation and 5 mW for THz detection. For THz generation the laser pulses are focused to a gallium arsenide substrate equipped with a photoconductive antenna, which is biased with a voltage of 125 V. For lock-in amplification the bias voltage is modulated at a frequency of 100 kHz. The emitted THz pulses, covering a spectral range from 10 GHz to about 1.5 THz, are imaged to the sample plane with 4 off-axis parabolic mirrors. The 550 μm thick silicon substrate with the split ring resonators on one surface are oriented with the bare surface facing the incident THz radiation. A few microns above the split ring resonator is the 100 μm thick zinc telluride (ZnTe) detection crystal with a [100] orientation. It is mounted on a 3 mm thick sapphire substrate to shift all etalon replica well outside the detection time window. The optical readout pulse comes from the opposite side and is focused through the sapphire plate at the ZnTe surface facing the split ring resonator. This surface is coated with a highly reflective multilayer system and reflects the optical readout pulse so that it propagates

collinearly with the THz pulse on its way back through the ZnTe crystal. The size of the focus is approximately 20 μm, which determines the lateral spatial resolution of the THz detection unit, and the Rayleigh length is 395 μm. Raster-scanning the detection crystal together with the optical readout pulse horizontally and vertically allows to map out the transverse spatial distribution of the electric field component normal to the plane containing the split ring resonator. The spatial increments match the spatial resolution of 20 μm. The back reflected readout pulse is analyzed through a combination of a quarter-wave plate, a polarization beam splitter, and a balanced photo-detector. The electric signal is amplified by a lock-in amplifier and transmitted to a computer for further processing. The measured signal is directly proportional to the instantaneous electric field of the THz waveform, which temporally overlaps with the optical readout pulse. To map out the entire time dependent THz waveform the readout pulse is scanned across the waveform in increments of 66 fs in a time window of 66 ps.

Simulations: The numerical simulations are based on the finite element method (FEM) using a commercial software package[1]. We perform frequency dependent modeling to determine the spatial distribution of all relevant quantities, such as the charge density, the electric field or the electric field enhancement. The frequency dependent approach has the advantage that the dispersive properties of the materials can easily be included. For gold we use a complex conductivity as obtained from the Drude model with the values for the plasma frequency and the damping constant taken from reference[2]. Even though thin metallic films have been shown to exhibit a smaller conductivity than the corresponding bulk materials we observe that the effect of even a tenfold increased damping constant on the electric field enhancement is negligible. The refractive indices of the silicon substrate and ZnTe detection crystal are 3.41[3] and 3.183[4], respectively.

The split ring resonator is positioned in the center of a rectangular box-shaped simulation domain (1 x 1 x 1 mm^3). As shown in Fig. S1a), the split ring resonator is placed on a 550 μm thick high resistivity silicon wafer and the distance between the silicon substrate and the 100 μm thick ZnTe crystal is D = 2 μm. The simulation domain is discretized using a tetrahedral mesh and additional mesh layers are added at all metallic interfaces. A plane harmonic wave is launched from the surface opposite to the plane containing the split ring resonator and the frequency is scanned parametrically between 10 GHz and 1 THz. To all other surfaces scattering boundary conditions are applied. Resonances occur whenever the length of the unfolded split ring resonator corresponds to integer multiples of half the wavelength. Due to

the symmetry of the modes relative to the linearly polarized incident THz pulse odd-numbered resonances are excited when the electric field of the THz pulse is parallel and even-numbered when it is polarized perpendicular to the gap[5]. Here, we consider electric field coupling, that is, the electric field is parallel to the side containing the gap and the *E*, *H*, and *k* triad of the incoming THz field is oriented along the *x*, *y* and *z* axes.

From the simulations, we first extract the frequency dependent response of the split ring resonator by analyzing the far-field transmission. Of specific interest are the first and the third order resonance frequencies, ν_1 and ν_3, which occur at 60 GHz and 185 GHz, 55 GHz and 180 GHz, and 50 GHz and 175 GHz for gap widths of 970 nm, 500 nm, and 100 nm, respectively. At the third order resonance frequency we then map out the spatial distribution of the electric field component normal to the plane containing the split ring resonator, i.e. $E_z(x,y,z_j,\nu_3)$, in form of two-dimensional intensity graphs of its amplitude at various heights z_j above the resonator, as shown in Fig. S1b).

Finally, we extract from the simulations the electric field averaged over the gap volume. Dividing this averaged field value by the value averaged over the same volume but without the split ring resonator present yields the electric field enhancement. The same procedure was applied to extract the magnetic field enhancement.

Data analysis and electric field calibration: The determination the electric field enhancement and specifically the time dependent electric field in the gap relies on an indirect method as there exists no detector with sufficient spatial resolution to measure these quantities directly. Our method is based on a numerical model which simulates the linear electromagnetic properties of the split ring resonator sandwiched in between the substrate and the detection crystal as outlined above. It predicts 1) the measured electric field $E_x(t)=\gamma_1 E_{x0}(t)$ without the split ring resonator present, 2) the normal electric field distribution $E_z(x,y,t) = \gamma_2(x,y) E_{x0}(t)$ and 3) the in-gap electric field $E_{gap,x}(t)=\gamma_3 E_{x0}(t)$, with the incident field $E_{x0}(t)$ and the constants γ_1, $\gamma_2(x,y)$, γ_3 being a result of the simulations. First, we determine $E_{0x}(t)= E_x(t)/\gamma_1$ through a reference measurement and then we simulate $E_z(x,y,t) = \gamma_2(x,y)/\gamma_1 E_x(t)$. Next we measure the spatially resolved out-of-plane electric field, and thus the charge distribution on the split ring resonator which is responsible for the capacitive charging of the gap. The argument now is that if the model succeeds to predict the measured out-of-plane electric field distribution it will also correctly predict the in-gap electric field and the electric field enhancement.

This multistep procedure uses the following recipe (the individual steps are explained in detail in the subsections hereafter):

I. Absolute calibration of the measured time dependent electric field distribution $E_z(x,y,t)$ based on the parameters of the detection unit.

II. Fine-tuning the distance between the split ring resonator and the detection crystal in the simulations by comparing the simulated to the measured resonance frequencies.

III. Identification of the effective height z_j at which the best correlation between the measured $|E_z(x,y,\nu_3)|$ and the simulated electric field distribution $|E_z(x,y,z_j,\nu_3)|$ is found.

IV. Determination of the phase associated to the electric field enhancement versus frequency by means of Kramers-Kronig's relation and the averaged time dependent electric field $E_{gap,x}(t)$ in the gap.

I. We start by assigning an absolute electric field scale to the measured electric field distribution $E_z(x,y,t)$. In the balanced electro-optic detection scheme the average power of the probe light incident on the two photodiodes is given by

$$\begin{aligned} P_x(x,y,t) &= \frac{P_0}{2}\left[1+\sin\Delta\varphi(x,y,t)\right] \\ P_y(x,y,t) &= \frac{P_0}{2}\left[1-\sin\Delta\varphi(x,y,t)\right] \end{aligned} \qquad \text{(S.1)}$$

with the THz-induced instantaneous phase shift $\Delta\varphi(x,y,t)$ and the total average power of the probe P_0 [6]. When no THz field is present the average power levels are balanced, i.e.

$$P_{x0} = P_{y0} = \frac{P_0}{2} \qquad \text{(S.2)}$$

The induced phase shift is proportional to the THz electric field

$$\Delta\varphi(x,y,t) = \frac{\omega L}{c} n_o^3 r_{41} E_z(x,y,t) \qquad \text{(S.3)}$$

where ω is the angular baseband frequency of the probe field, L the thickness of the ZnTe detection crystal, c the speed of light in vacuum, n_o the ordinary refractive index of ZnTe, and r_{41} the relevant electro-optic tensor component. Equation (S.3) is valid for ideal phase-matching, i.e. for thin enough ZnTe crystals, which is the case here. Since the Rayleigh length of the probe beam is approximately four times the ZnTe crystal thickness, we assume that the measured THz electric field is $E_z(x,y,t) = \frac{1}{L}\int_D^{D+L} dz\, E_z(x,y,z,t)$, with the distance between the split ring resonator and the detection crystal D. Thus, for small phase shifts we find

$$E_z(x,y,t) = \frac{c}{\omega L n_0^3 r_{41}} \frac{\Delta P(x,y,t)}{2P_{x0}} \qquad \text{(S.4)}$$

In the balanced detection unit (Thorlabs PDB210A) the average power P_{x0} can be measured at the monitor output and the voltage detected is

$$U_{x0} = G\, P_{x0} \qquad \text{(S.5)}$$

with the calibration constant $G = 9.18\cdot 10^3$ V/W. The difference voltage is pre-amplified before being measured by a lock-in amplifier and is given by

$$\Delta U(x,y,t) = \gamma\, \Delta P(x,y,t) \qquad \text{(S.6)}$$

with the calibration constant $\gamma = 9.8\cdot 10^4$ V/W. With this we find

$$E_z(x,y,t) = \frac{c}{\omega L n_0^3 r_{41}} \frac{G}{2\gamma} \frac{\Delta U(x,y,t)}{U_{x0}} = 6.2594\cdot 10^5 \frac{\Delta U(x,y,t)}{U_{x0}} \frac{\text{V}}{\text{m}} \qquad \text{(S.7)}$$

where we have used $c = 2.998\cdot 10^8$ m/s, $\omega = 2.415\cdot 10^{15}$ rad/s, $L = 100$ µm, $n_o = 2.853$, and $r_{41} = 4\cdot 10^{-12}$ m/V. That is, from a measurement of ΔU and U_{x0} we can determine the calibrated instantaneous THz electric field $E_z(x,y,t)$.

II. Next, we fine-tune the distance D between the split ring resonator and the detection crystal in the simulations by comparing the simulated to the measured resonance

frequencies. The distance influences the effective dielectric constant of the composite material (silicon, air, and ZnTe) surrounding the split ring resonator and thereby the resonance frequencies. Experimentally, we extract the resonance frequencies from the Fourier transform $E_z(x_0,y_0,\nu)$ at a suitable position (x_0,y_0) on the split ring resonator. The best agreement between experiment and simulation is found for distance of $D = 2$ μm, which agrees approximately with optical inspection from the side.

III. We then determine the height z_0 at which the simulated electric field distribution $E_z(x,y,z_0,\nu_3) = \frac{1}{L}\int_D^{D+L} dz\, E_z(x,y,z,\nu_3)$ (see Fig. S1) at the third resonance correlates best with the measured electric field distribution, following a procedure suggested in reference[7]. The best correlation is found at approximately $z_0 = 40$ μm and Fig. S2 shows that the correlation is close to perfect for all three gap widths examined.

IV. To calculate the time dependent electric field in the gap, we first use the Kramers-Kronig's relation to determine the frequency dependent phase corresponding to the absolute value of the electric field enhancement shown in Fig. 4a). We then multiply the incident spectral electric field by the complex electric field enhancement and apply an inverse Fourier transformation to obtain $E_{gap,x}(t)$ (see Fig. 4b).

Accounting for all errors, i.e. of the distance between the substrate and the ZnTe crystal, (2 ± 2) μm, of the height at which the electric field is considered, (40 ± 5) μm, and of the different calibration constants, yields relative errors for the in-gap electric field and the field enhancement of about ±21% and ±30%, respectively.

References

[1] Jin, J. The Finite Element Method in Electromagnetics. *Willey-IEEE Press*, Second ed., 2002.

[2] Ordal, M. A., Bell, R. J., Jr, R. W. A., Long, L. L. & Querry, M. R. Optical properties of fourteen metals in the infrared and far infrared: Al, Co, Cu, Au, Fe, Pb, Mo, Ni, Pd, Pt, Ag, Ti, V, and W. *Appl. Opt.* **24**, 4493-4499 (1985).

[3] Palik, E.D. Handbook of optical constants of solids, *Elsevier Science*, 1985.

[4] Tripathi, S.R., Aoki, M., Asahi, T., Hosako, I., Hiromoto, N., Accurate optical constants of ZnTe measured by THz-TDS with their standard deviations. *35th International Conference on Infrared Millimeter and Terahertz Waves*, 10.1109/ICIMW.2010.5613015 (2010).
[5] Bitzer, A., Merbold, H., Thoman, A., Feurer, T., Helm, H., & Walther, M. Terahertz near-field imaging of electric and magnetic resonances of a planar metamaterial, *Opt. Express* **17**, 3826-3834 (2009).
[6] Lee, Y-S., Principles of Terahertz Science and Technology, *Springer,* New York, 2009.
[7] Seo, M. A., Adam, A. J. L., Kang, J. H., Lee, J. W., Ahn, K. J., Park, Q. H., Planken, P. C. M., Kim, D. S. Near field imaging of terahertz focusing onto rectangular apertures. *Opt. Express* **16**, 20485-20489 (2008).

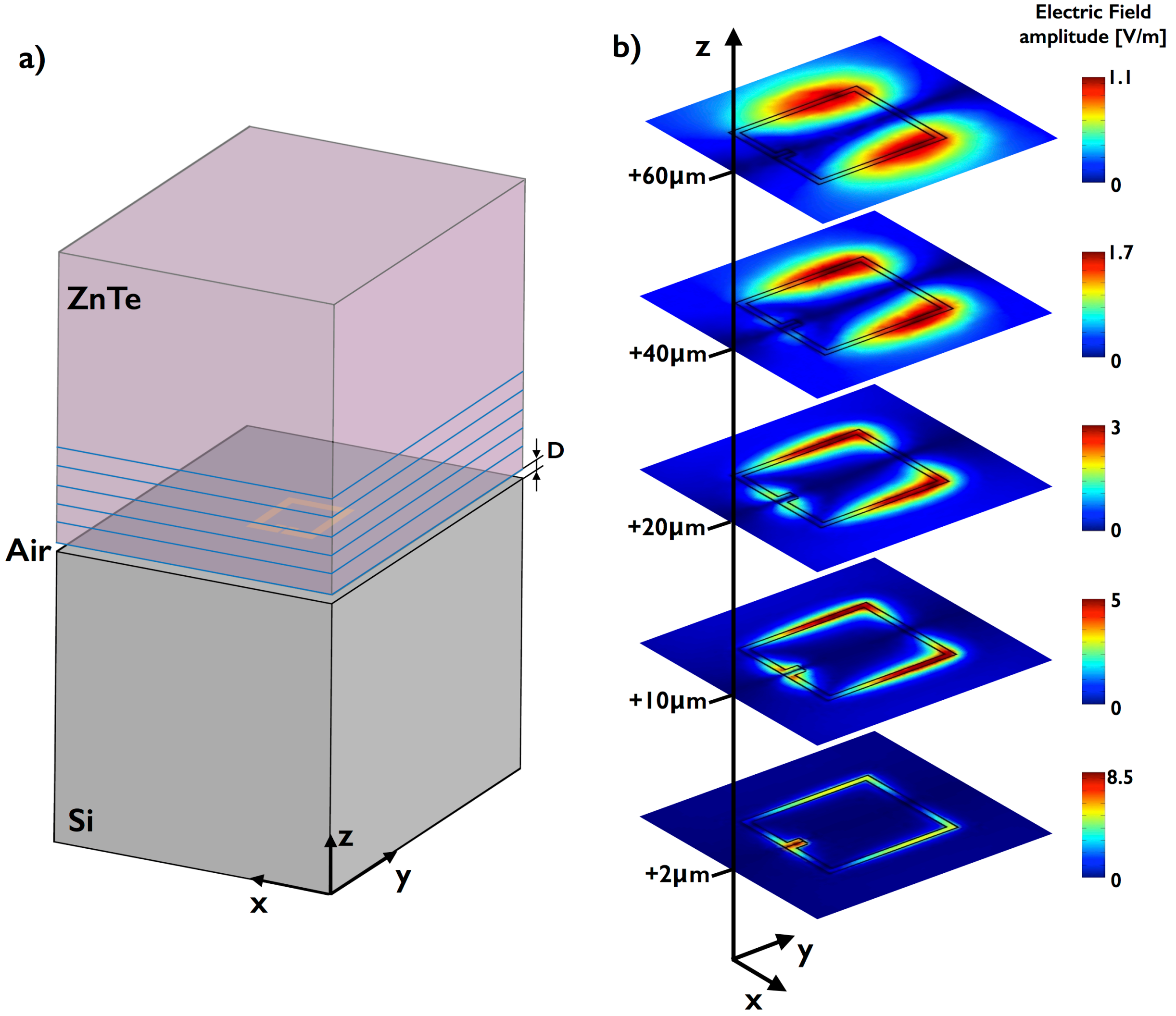

Figure S1: a) The simulation domain showing the silicon substrate, the split ring resonator, and the ZnTe detection crystal. b) Two-dimensional distributions of $|E_z(x,y,z_j,\nu_3)|$ in different xy-planes above the split ring resonator, i.e. at distances of z_j = 2, 10, 20, 40, and 60 µm as indicated in a).

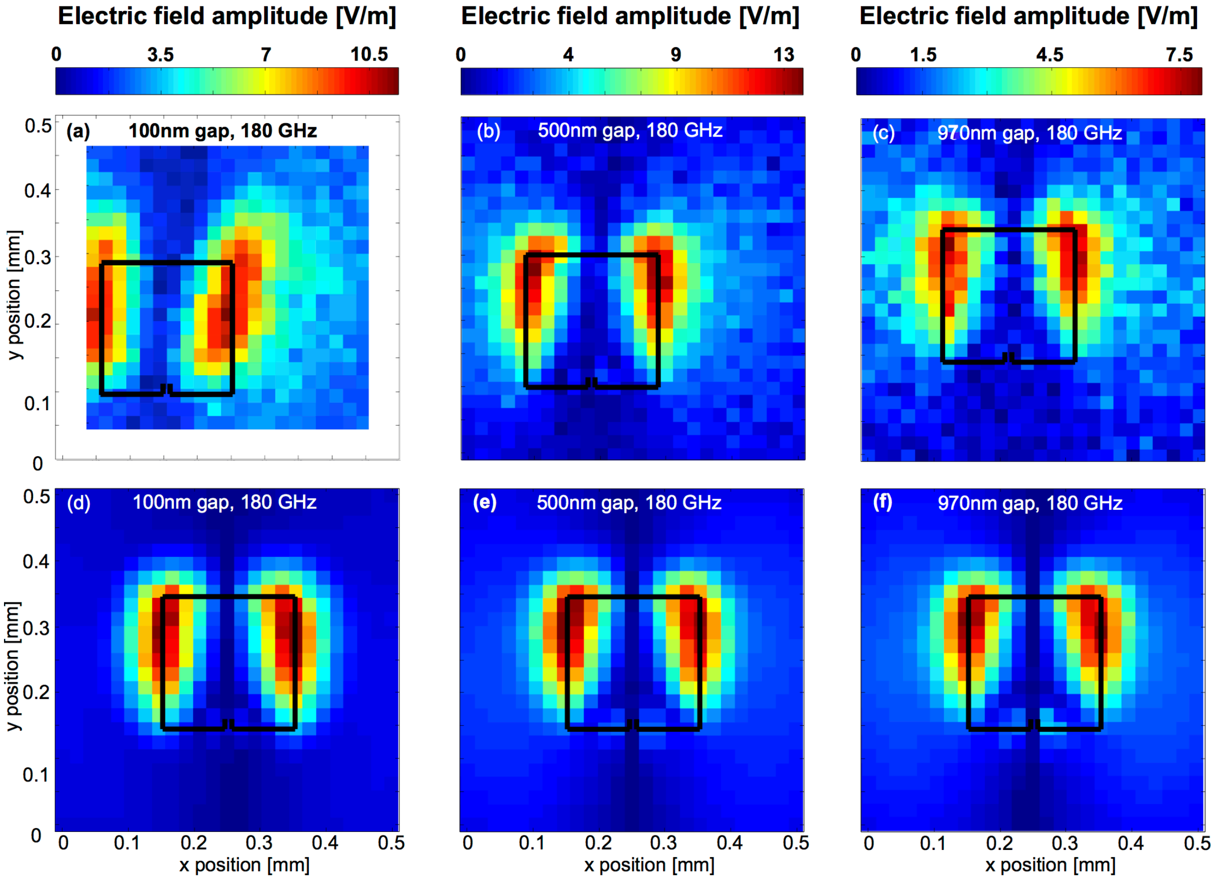

Figure S2: Intensity graphs of the measured (a – c) and the simulated (d – f) out-of-plane electric field amplitude distribution at the second resonance ν_3. Three different split ring resonators with a 100 nm (a, d), a 500 nm (b, e), and a 970 nm (c, f) wide gap are shown.
